# Artificial intelligence-enhanced electrocardiography for early assessment of coronavirus disease 2019 severity

**DOI:** 10.1038/s41598-023-42252-5

**Published:** 2023-09-13

**Authors:** Yong-Soo Baek, Yoonsu Jo, Sang-Chul Lee, Wonik Choi, Dae-Hyeok Kim

**Affiliations:** 1 Division of Cardiology, Department of Internal Medicine, Inha University College of Medicine, Inha University Hospital, 27 Inhang-ro, Jung-gu, Incheon, 22332 Republic of Korea; 2 School of Computer Science, University of Birmingham, Birmingham, B15 2TT UK; 3 DeepCardio Inc., 100 Inha-ro, Incheon, 22212 Republic of Korea; 4 Department of Computer Engineering, Inha University, 100 Inha-ro, Incheon, 22212 Republic of Korea; 5 Department of Information and Communication Engineering, Inha University, 100 Inha-ro, Michuhol-gu, Incheon, 22212 Republic of Korea

**Keywords:** Cardiology, Diseases, Medical research

## Abstract

Despite challenges in severity scoring systems, artificial intelligence-enhanced electrocardiography (AI-ECG) could assist in early coronavirus disease 2019 (COVID-19) severity prediction. Between March 2020 and June 2022, we enrolled 1453 COVID-19 patients (mean age: 59.7 ± 20.1 years; 54.2% male) who underwent ECGs at our emergency department before severity classification. The AI-ECG algorithm was evaluated for severity assessment during admission, compared to the Early Warning Scores (EWSs) using the area under the curve (AUC) of the receiver operating characteristic curve, precision, recall, and F1 score. During the internal and external validation, the AI algorithm demonstrated reasonable outcomes in predicting COVID-19 severity with AUCs of 0.735 (95% CI: 0.662–0.807) and 0.734 (95% CI: 0.688–0.781). Combined with EWSs, it showed reliable performance with an AUC of 0.833 (95% CI: 0.830–0.835), precision of 0.764 (95% CI: 0.757–0.771), recall of 0.747 (95% CI: 0.741–0.753), and F1 score of 0.747 (95% CI: 0.741–0.753). In Cox proportional hazards models, the AI-ECG revealed a significantly higher hazard ratio (HR, 2.019; 95% CI: 1.156–3.525, *p* = 0.014) for mortality, even after adjusting for relevant parameters. Therefore, application of AI-ECG has the potential to assist in early COVID-19 severity prediction, leading to improved patient management.

## Introduction

Coronavirus disease 2019 (COVID-19), caused by the severe acute respiratory syndrome coronavirus 2 (SARS-CoV-2), has become a pandemic with widespread increased mortality^1^. The spectrum of COVID-19 severity is broad and ranges from an asymptomatic and mild presentation to severe and critical illness^2–5^. There is increasing awareness of the cardiovascular manifestations of COVID-19 and their adverse effects on disease prognosis^6^. Acute cardiac injury has been reported in 8–62% of patients hospitalized with COVID-19 and is associated with greater disease severity, including the need for mechanical ventilation and death^7–9^.

Because of rapid fluctuation in infection rates and limitations in medical systems, the demand for tertiary medical services has increased. However, it is incredibly difficult to identify whether patients have good or poor prognoses in the initial stage, especially when patients are treated at home. Therefore, the early prediction of disease severity and prognosis has an important effect on clinical outcomes in patients with COVID-19.

SARS-CoV-2 infections result in electrocardiographic changes that enable the use of artificial intelligence-enhanced electrocardiography (AI-ECG) as a rapid screening test with a high negative predictive value^2^. Thus, AI-ECG may become a leading tool to assess the extent of cardiac involvement in patients with COVID-19, owing to its low cost, the feasibility of point-of-care testing, and the possibility of remote evaluations^10^. COVID-19 results in recognizable changes in the AI-ECG, and the absence of these changes exclude the presence of acute coronavirus infections, facilitating point-of-care screening.

The rapid influx of COVID-19 hospitalizations has placed a heavy load on the limited healthcare system; therefore, an efficient and streamlined risk stratification tool is required to predict the prognosis of patients. In this study, we aimed to assess whether AI using initial 12-lead ECGs could assist in the early prediction of COVID-19 disease severity.

## Methods

### Study population

**Figure 1:**
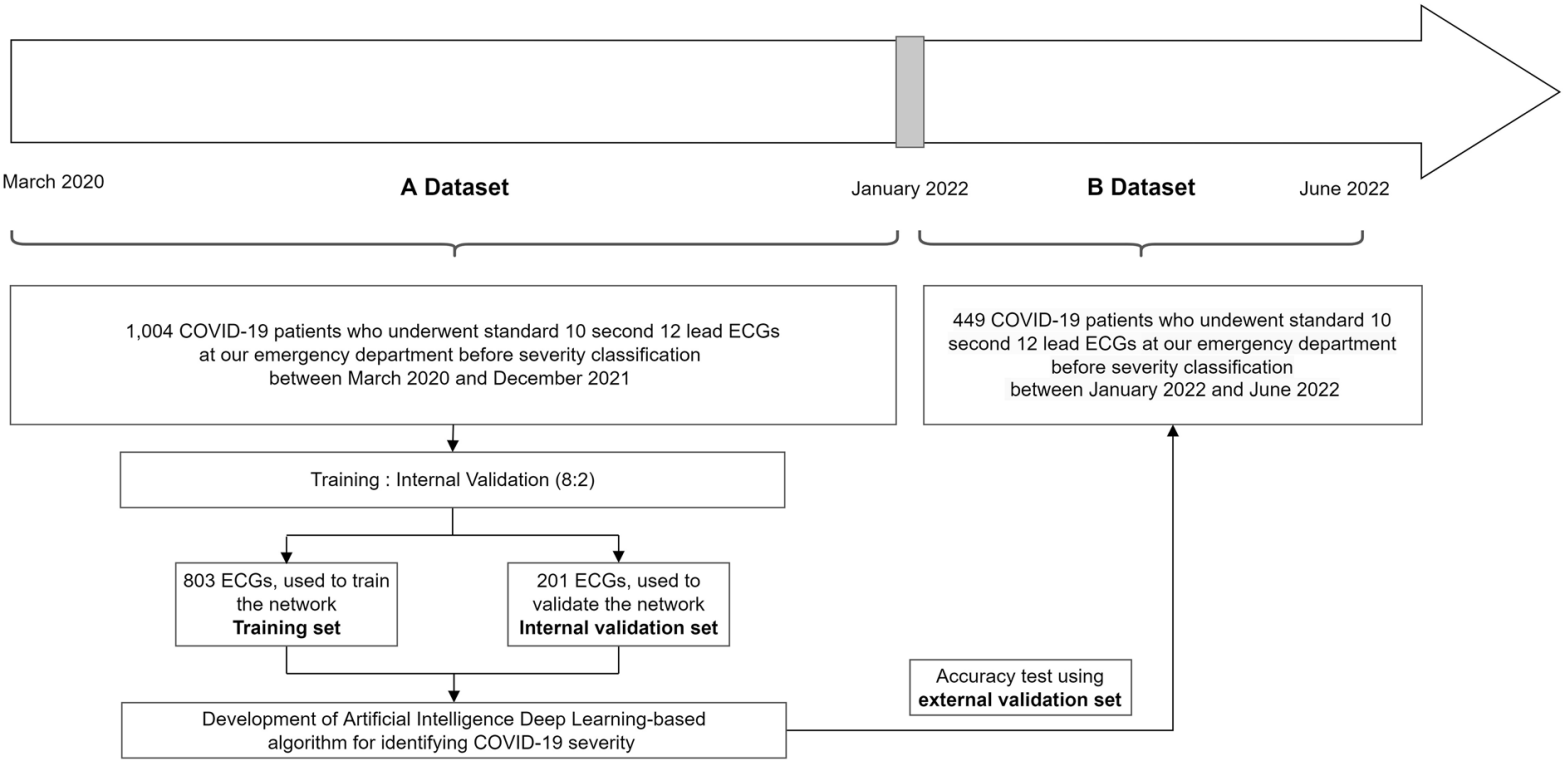
Study flow diagram showing the selection of patients with COVID-19 and the creation of the study datasets. ECGs were allocated to the training, internal validation, and external validation datasets using Data A and B. *ECG* electrocardiography; *COVID-19* coronavirus disease 2019.

We enrolled 1,453 adult patients (aged ≥ 18 years) who were diagnosed with COVID-19 and admitted to our tertiary hospital between March 2020 and June 2022. COVID-19 was diagnosed if the patient had a positive result in the SARS-CoV-2 polymerase chain reaction test. We included patients with comprehensive data, encompassing a 12-lead ECG indicating sinus rhythm, laboratory parameters, oxygen requirement status, clinical course, and outcomes. These patients had also undergone an ECG prior to any severity classification and before transitioning from the emergency department (ED) to COVID-19 dedicated wards, as our objective was to evaluate the predictive value of the AI algorithm using ECG data for early assessment of severity in COVID-19 patients. Patients with atrial fibrillation or atrial flutter were excluded due to their potential association with adverse clinical outcomes in COVID-19, which could introduce a confounding bias in prognosis prediction. We also excluded patients without 12-lead ECG data, those with discrepant admission data, those with an unclear discharge status, and those who had undergone ECG after transitioning from the ED to dedicated isolation rooms or the intensive care unit (ICU), implying their severity classification had already been determined. Dataset A, acquired from March 2020 to December 2021, was used for development and internal validation, and dataset B, acquired from January 2022 to June 2022 after the development of the AI, was used for external validation (Fig. 1).

The Institutional Review Board of the Inha University Hospital (2021-10-006) approved the study protocol and waived the need for informed consent owing to the impracticality of obtaining consent and the minimal harm resulting from the study. The study complied with the principles of the Declaration of Helsinki.

### COVID-19 severity classification

After transitioning from the ED to the COVID-19 dedicated wards, we classified patients based on the World Health Organization guideline into two categories: group 1 with mild-to-moderate illness, defined by not requiring oxygen therapy or low-flow oxygen therapy < 5 L via nasal prongs; and group 2 with severe-to-critical illness, characterized by the need of high-flow oxygen, continuous positive airway pressure, invasive mechanical ventilation, or extracorporeal membrane oxygenation [ECMO]^11–13^.

### Data collection and covariates

All data in the ECGs were acquired at a sampling rate of 500 Hz using a GE-Marquette ECG machine (General Electric Healthcare, Chicago, Illinois, United States). The raw data were stored as XML documents using the MUSE data management system in relational databases. All ECG data were manually adjudicated by two electrophysiologists. We included the demographic, laboratory, clinical, and ECG covariates in our prediction models. The demographic covariates included age, sex, ethnicity, and insurance type, and the vital signs included oxygen saturation, mean blood pressure, body temperature, and ventricular rate.

### AI algorithm model for predicting COVID-19 severity

The AI algorithm was developed using Long Short-Term Memory Fully Convolutional Networks (LSTM-FCN) to manage sequential data reflecting the ECG characteristics. With an attention mechanism, the AI algorithm can automatically capture the most important ECG characteristics and classify the data. We extracted and analyzed the XML data from the MUSE data management system, and to minimize the artifacts, all data files were stored in the XML format on a GE ECG machine (General Electric Healthcare, Chicago, Illinois, United States).

**Figure 2:**
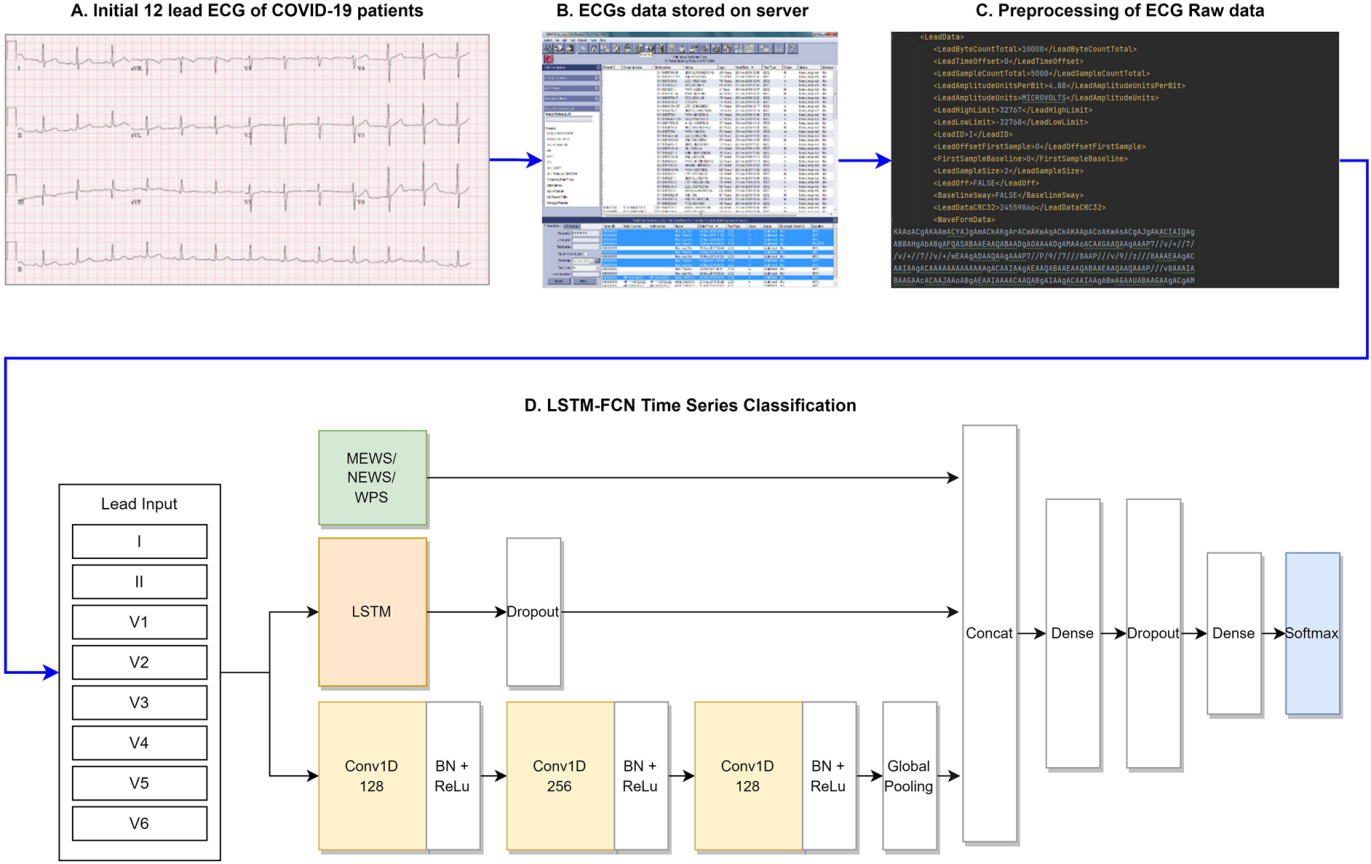
Description of the artificial intelligence algorithm for predicting the severity in patients with COVID-19. *COVID-19* coronavirus disease 2019.

The ECGs were originally recorded from 12 leads; however, because of the device’s data storage method, only data from eight leads were stored, excluding lead III, aVR, aVL, and aVF. Simple arithmetic operations can be used to calculate the data from those four leads, and it is common to apply these processes to approximate the data^14^. Therefore, only the eight recorded signals of leads I, II, V1, V2, V3, V4, V5, and V6 were used in this study. The signals from each lead were simultaneously measured for 10 s, and when the Base64-encoded value was read, eight one-dimensional arrays for each XML file were obtained. As a 10-s signal has multiple pulses and heart rate varies from person to person, we obtained approximately 10 or more pulses per person (Fig. 2). We specified the position of the P, QRS, and T waves and analyzed those waves separately to avoid any bias from the variable heart rate. We used an algorithm to detect the R peaks, and the P, QRS, and T waves were located afterwards. We then analyzed each wave using AI, which calculated the scores for each wave. The result was presented by calculating the mean score. Additionally, we utilized the Class Activation Map (CAM) to highlight ECG segments that indicate regions significantly contributing to the severity classification in COVID-19 patients^15^.

### Confirmation of the performance of AI-ECGs for predicting COVID-19 severity

We trained and validated the AI-enhanced ECGs to assess the severity of COVID-19 in patients who underwent their initial ECG at our ED before severity classification. We tested the accuracy of the AI-ECG using an external dataset. We compared the area under the receiver operating characteristic curve (AUROC) to confirm the accuracy of the developed AI-ECGs. AUROC was calculated using the AI-ECG in the presence of severe-to-critical illness in COVID-19 patients with the Early Warning Scores (EWSs), including the Modified Early Warning Score (MEWS), National Early Warning Score (NEWS), and the Worthing Physiological Scoring System (WPS). Those scores were calculated after relevant data assessment^16–18^.

### Statistical analysis

Continuous variables are reported as means ± standard deviations or medians and interquartile ranges, and categorical variables are presented as percentages and frequencies. Comparisons between groups were performed using the independent sample t-test or chi-square test. The performance of the AI model was measured using the AUROC to predict the dataset accuracy, recall (sensitivity), specificity, and F1 score. Recall is the ratio of correctly predicted positive observations to the total observations, while the F1 score (balanced F-score) is the harmonic mean of the precision and recall. In addition, to predict mortality in the admission of COVID-19 patients, we performed a Cox proportional-hazards model regression analysis. For all variables, *p* < 0·05 was considered statistically significant. Statistical analyses were performed using SPSS statistical software for Windows (version 21.0; IBM, Armonk, New York, United States).

## Results

### Patient characteristics

The baseline characteristics, comorbidities, and laboratory and electrocardiographic findings of the enrolled patients are shown in Table 1. The mean age of the 1,453 participants was 59.7 ± 20.1 years, and 54.2% of the patients were male. Group 1 (mild-to-moderate illness, with no need for oxygen therapy or low-flow oxygen therapy) included 892 patients, while group 2 (severe-to-critical illness and required higher treatment than high-flow oxygen [5 L via nasal prong]) included 561 patients. For both datasets A and B, the proportions of patients with hypertension (*p* < 0.001), diabetes mellitus (*p* < 0.001), and strokes (*p* < 0.001) were significantly greater in group 2 than in group 1. Regarding the laboratory findings, the white blood cell and platelet counts and C-reactive protein, N-terminal-pro hormone B-type natriuretic peptide, creatine phosphokinase, creatine kinase-MB, blood urea nitrogen, and serum creatinine levels were also higher in group 2 than in group 1 in datasets A and B. On comparing the ECG findings between the two groups, we found that the patients in group 2 had a higher heart rate, prolonged QRS duration, and longer corrected QT (QTc) interval than those in group 1.

**Table 1:** Patient characteristics and laboratory and electrocardiographic findings at enrollment.

	Dataset A				Dataset B				
	Overall	Mild-to-moderate illness	Severe-to-critical illness	*p-value	Overall	Mild-to-moderate illness	Severe-to-critical illness	*p-value	†*p*-value
	(n = 1,004)	(n = 689)	(n = 142)		(n = 449)	(n = 203)	(n = 246)		
Age, years	55.2 ± 19.7	50.6 ± 18.3	64.7 ± 18.4	< 0.001	69.9 ± 17.6	63.3 ± 18.8	75.4 ± 13.7	< 0.001	< 0.001
Male, n (%)	566 (56.4)	430 (62.4)	136 (43.2)	< 0.001	221(49.2)	116 (57.1)	105 (42.7)	0.002	0.012
Body mass index, kg/m2	24.5 ± 4.7	24.3 ± 4.3	24.7 ± 5.4	0.249	22.1 ± 4.9	23.1 ± 4.5	21.3 ± 5.0	< 0.001	< 0.001
Hypertension, n (%)	368 (36.7)	191 (27.7)	177 (56.2)	< 0.001	242 (53.9)	103 (50.7)	139 (56.5)	0.223	< 0.001
Diabetes mellitus, n (%)	208 (20.7)	115 (16.7)	93 (29.5)	< 0.001	157 (43.0)	61 (30.0)	96 (39.0)	0.047	< 0.001
Heart failure, n (%)	13 (1.3)	8 (1.2)	5 (1.6)	0.579	29 (6.5)	4 (2.0)	25 (10.2)	< 0.001	< 0.001
Stroke, n (%)	62 (6.2)	31 (4.5)	31 (9.8)	0.001	64 (14.3)	24 (11.8)	40 (62.5)	0.222	< 0.001
Vascular disease, n (%)	60 (6.0)	43 (6.2)	17 (5.4)	0.668	81 (18.0)	26 (12.8)	55 (22.4)	0.01	< 0.001
Initial vital sign									
SBP, mmHg	133.3 ± 21.6	132.7 ± 21.3	134.3 ± 22.2	0.306	133.3 ± 26.7	137.2 ± 23.4	130.1 ± 28.7	0.005	0.963
DBP, mmHg	79.4 ± 11.9	80.1 ± 11.9	77.5 ± 11.4	0.001	76.7 ± 15.9	80.8 ± 13.8	73.2 ± 16.5	< 0.001	< 0.001
HR, /min	87.5 ± 17.6	87.5 ± 16.3	87.2 ± 17.6	0.743	89.1 ± 18.3	87.1 ± 16.6	91.1 ± 19.3	0.023	0.06
RR, /min	20.5 ± 3.1	19.5 ± 1.8	21.0 ± 4.1	< 0.001	20.2 ± 4.1	19.2 ± 2.1	20.7 ± 4.1	< 0.001	0.98
Body Temperature, °C	37.2 ± 0.8	37.2 ± 0.7	37.2 ± 0.9	0.307	36.8 ± 0.7	36.9 ± 0.6	36.7 ± 0.8	0.018	< 0.001
Laboratory findings									
WBC, k/μL	5.94 ± 3.06	5.38 ± 2.45	7.16 ± 3.81	< 0.001	9.45 ± 5.77	8.01 ± 3.85	10.6 ± 6.74	< 0.001	< 0.001
Hb, g/dL	13.49 ± 1.87	13.58 ± 1.80	13.30 ± 2.01	0.031	11.57 ± 2.74	11.61 ± 2.73	11.54 ± 2.76	0.798	< 0.001
PLT, k/μL	203.9 ± 82.8	204.7 ± 72.2	202.1 ± 102.4	0.647	234.3 ± 116.2	234.2 ± 105.9	234.4 ± 124.1	0.988	< 0.001
CRP, mg/dL	4.01 ± 15.81	2.28 ± 3.81	7.80 ± 27.27	< 0.001	6.56 ± 8.27	3.85 ± 5.66	8.73 ± 9.33	< 0.001	0.001
NT-proBNP, pg/mL	1146.8 ± 4730.9	525.4 ± 3385.4	2015.6 ± 6038.5	< 0.001	5045.5 ± 8729.5	2770.2 ± 7248.6	6262.2 ± 9213.2	< 0.001	< 0.001
CK-MB, ng/mL	1.80 ± 4.21	1.19 ± 1.00	3.10 ± 7.13	< 0.001	6.6 ± 20.3	2.78 ± 4.23	9.17 ± 25.6	< 0.001	< 0.001
Troponin-I,	0.22 ± 0.77	0.15 ± 0.03	0.33 ± 1.28	0.002	0.48 ± 1.78	0.17 ± 0.05	0.73 ± 2.34	0.003	0.005
BUN, mg/dL	15.4 ± 12.3	12.6 ± 7.7	21.4 ± 17.2	< 0.001	27.6 ± 25.4	20.2 ± 16.4	33.6 ± 29.6	< 0.001	< 0.001
Creatinine, mg/dL	1.01 ± 1.48	0.90 ± 1.40	1.25 ± 1.61	0.001	1.52 ± 1.82	1.22 ± 1.42	1.76 ± 2.06	0.002	< 0.001
pH (ABGA via O2)	7.43 ± 0.74	7.43 ± 0.54	7.42 ± 0.08	0.768	7.40 ± 0.12	7.43 ± 0.06	7.38 ± 0.14	< 0.001	< 0.001
PCO2 (ABGA via O2), mmHg	35.5 ± 6.7	36.0 ± 5.7	35.1 ± 7.4	0.139	38.4 ± 5.8	35.3 ± 5.8	40.1 ± 28.8	0.016	0.023
PO2 (ABGA via O2), mmHg	91.2 ± 51.6	88.3 ± 30.5	93.3 ± 62.3	0.34	86.1 ± 43.2	84.1 ± 26.3	87.1 ± 49.8	0.535	0.143
ECG findings									
Heart rate, bpm	80.6 ± 16.8	79 ± 15.1	84.1 ± 19.5	0.00002	91.9 ± 22.3	86 ± 18.1	97.6 ± 24.5	< 0.00001	< 0.00001
PR interval, ms	158.7 ± 26.1	158.5 ± 25.2	159.3 ± 28	0.68745	157.6 ± 27.6	160 ± 26.6	155 ± 28.4	0.10316	0.51654
QRS duration, ms	89.3 ± 13.6	88.7 ± 13	90.6 ± 14.7	0.05768	88.7 ± 17.9	88.2 ± 15.1	89.2 ± 20.3	0.58665	0.53396
QT interval, ms	383.8 ± 39.1	383.1 ± 33.9	385.3 ± 48.6	0.447	377.1 ± 50.8	380.5 ± 41.3	373.9 ± 58.4	0.21109	0.01224
QTc, ms	438.2 ± 29.9	433.8 ± 24.5	447.7 ± 37.5	< 0.00001	457.1 ± 38.4	448.4 ± 30.6	465.5 ± 43.1	0.00002	< 0.00001
PAxis	46 ± 22.9	47 ± 22.3	43.5 ± 23.9	0.04232	50.8 ± 24.2	49.9 ± 21.5	51.8 ± 26.9	0.47994	0.00178
RAxis	37.2 ± 39.4	39.7 ± 39.8	31.8 ± 38.1	0.00589	32 ± 46.5	34.1 ± 39.7	30 ± 52.3	0.39827	0.04506
TAxis	40.2 ± 37.8	39.7 ± 34.3	41.3 ± 44.4	0.57288	50.1 ± 52.5	41.8 ± 38	58.3 ± 62.5	0.00263	0.00018

Values are expressed as the n (%) or means ± standard deviations.

*p-value of the student’s t-test or chi-square test between the mild-to-moderate and severe-to-critical illness groups.

^†^*p*-value of the student’s t-test or chi-square test between the datasets A and B.

bpm: beats per minute; CHA2DS2-VASc: a score taking into account congestive heart failure, hypertension, age ≥ 75 years, diabetes mellitus, previous stroke/transient ischemic attack, vascular disease, age 65–74 years, and sex (female); ECG: electrocardiography; PAF: paroxysmal atrial fibrillation; QRSd: QRS duration; TIA: transient ischemic attack; SBP, systolic blood pressure; DBP, diastolic blood pressure; HR, heart rate; RR, respiratory rate; WBC, white blood cells, Hb, hemoglobin; PLT, platelets; CRP, C-reactive protein; NT-proBNP, N-terminal pro-brain natriuretic peptide; CK-MB, Creatine kinase-MB; BUN, blood urea nitrogen.

### Clinical outcomes and the EWS according to the COVID-19 classification

The in-hospital mortality rate was 8.3% (121 patients), and all patients belonged to group 2. The proportions of heart failure, intensive care unit care, invasive mechanical ventilation, and ECMO were significantly higher in group 2 than in group 1 (*p* < 0.001). Overall, the duration of hospitalization was significantly longer in group 2 than in group 1 (*p* < 0.001; Table 2). In both datasets A and B, the MEWS, NEWS and WPS scores were significantly higher in group 2 than in group 1 (*p* < 0.001; Table 3).

**Table 2:** Clinical outcomes according to the COVID-19 classification.

	Dataset A				Dataset B				
	Overall	Mild-to-moderate illness	Severe-to-critical illness	*p-value	Overall	Mild-to-moderate illness	Severe-to-critical illness	*p-value	†*p*-value
	(n = 1,004)	(n = 689)	(n = 142)		(n = 449)	(n = 203)	(n = 246)		
Heart failure, n (%)	207 (20.6)	34 (4.9)	173 (54.9)	< 0.001	189 (42.1)	75 (36.9)	114 (46.3)	0.045	< 0.001
ICU care, n (%)	128 (12.7)	6 (0.9)	122 (38.7)	< 0.001	191 (42.5)	7 (3.4)	184 (74.8)	< 0.001	< 0.001
Invasive mechanical ventilation, n (%)	93 (9.3)	0 (0)	93 (29.5)	< 0.001	61(13.6)	2 (1.0)	59 (24.0)	< 0.001	0.016
ECMO, n (%)	31 (3.1)	0 (0)	31 (9.8)	< 0.001	4 (0.9)	0 (0)	4 (1.6)	0.068	0.015
Total admission duration, days	18.2 ± 16.8	13.3 ± 6.4	28.7 ± 25.4	< 0.001	16.6 ± 17.1	10.8 ± 8.6	21.1 ± 20.5	< 0.001	0.098
In-hospital mortality, n (%)	45 (4.5)	0 (0)	45 (14.3)	< 0.001	76 (16.9)	0 (0)	76 (30.9)	< 0.001	< 0.001

Values are expressed as the n (%) or means ± standard deviations.

ICU, intensive care unit; ECMO, extracorporeal membrane oxygenation.

*p-value of Student’s t-test or chi-square test between group 1 and group 2.

**Table 3:** A comparison among the Modified Early Warning Score, National Early Warning Score, and Worthing Physiological Scoring System according to disease severity in patients with COVID-19.

	Dataset A				Dataset B				
	Overall	Mild-to-moderate illness	Severe-to-critical illness	*p-value	Overall	Mild-to-moderate illness	Severe-to-critical illness	*p-value	†*p*-value
	(n = 1,004)	(n = 689)	(n = 142)		(n = 449)	(n = 203)	(n = 246)		
Modified Early Warning Score (MEWS)	1.9 ± 1.2	1.7 ± 0.9	2.3 ± 1.5	< 0.001	2.5 ± 1.8	1.7 ± 1.1	3.1 ± 2.0	< 0.001	< 0.001
National Early Warning Score (NEWS)	2.2 ± 2.4	1.6 ± 1.7	3.6 ± 2.9	< 0.001	4.0 ± 3.4	2.0 ± 2.2	5.6 ± 3.4	< 0.001	< 0.001
Worthing Physiological Scoring System (WPS)	1.7 ± 1.6	1.3 ± 1.1	2.6 ± 2.0	< 0.001	3.1 ± 2.6	1.7 ± 1.8	4.3 ± 2.6	< 0.001	< 0.001

Values are expressed as the n (%) or means ± standard deviations.

*p-value of the Student’s *t* test or chi-square test between group 1 and group 2.

### Performance of the AI model for predicting the severity and prognosis of COVID-19

**Figure 3:**
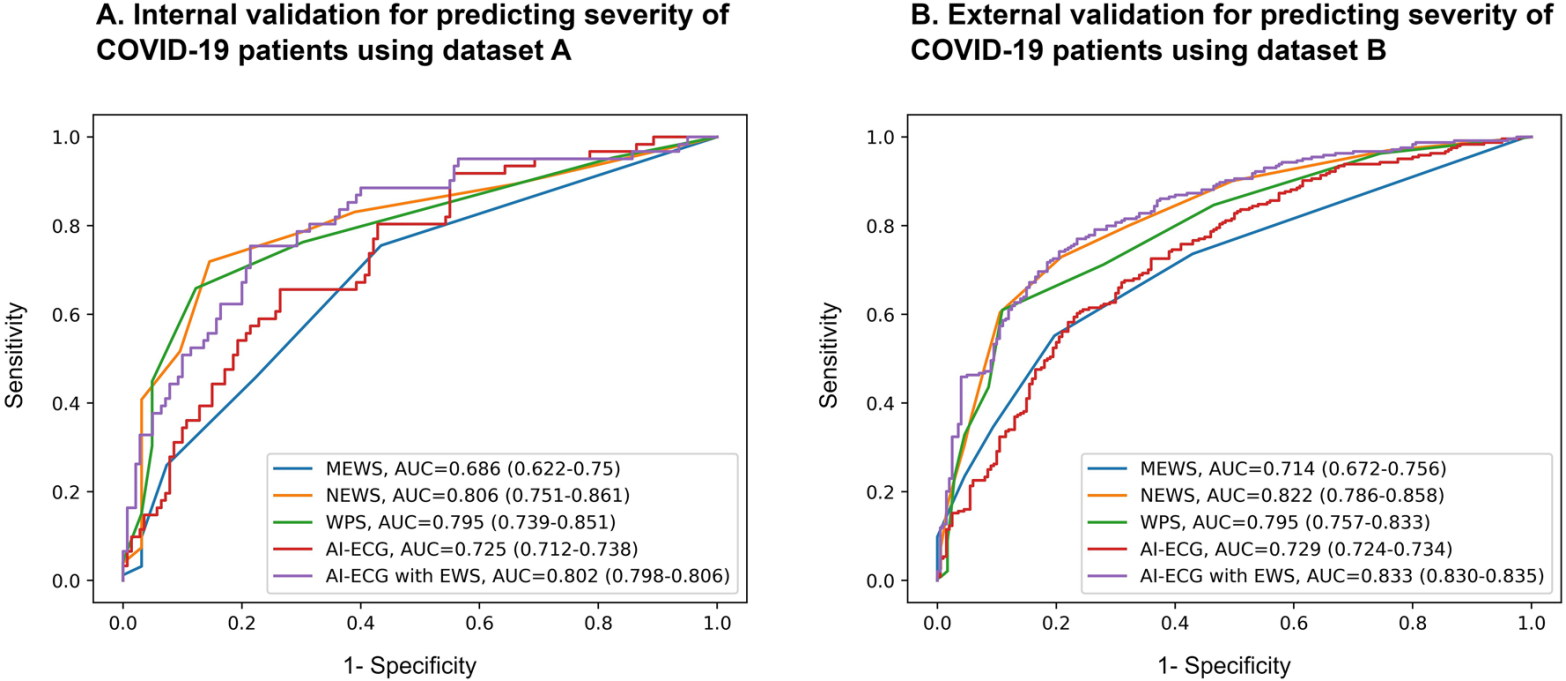
Multiclass ROC curves with deep neural networks. (**A**) Internal validation for predicting the severity of COVID-19 patients using dataset A. (**B**) External validation for predicting the severity of COVID-19 patients using dataset A. *COVID-19* coronavirus disease 2019; *ROC* receiver operating characteristic.

During the internal and external validations, the AUCs of the AI model for predicting severe-to-critical illness in patients with COVID-19 were 0.725 (95% CI: 0.712–0.738) and 0.729 (95% CI: 0.724–0.734), respectively (Fig. 3A,B; Table 4). During the external validation, the AUCs of the MEWS, NEWS, and WPS for detecting severe-to-critical illness in patients with COVID-19 were 0.714 (95% CI: 0.672–0.756), 0.822 (95% CI: 0.786–0.858), and 0.795 (95% CI: 0.757–0.833), respectively. As shown in Fig. 3, the AI tool combined with the EWS showed reliable performance for predicting patients with severe-to critical COVID-19 with an AUC of 0.802 (95% CI: 0.798–0.806) during internal validation and 0.833 (95% CI: 0.830–0.835) during external validation.

**Table 4:** AI model performance for predicting COVID-19 severity in hospitalized patients.

	Internal validation				External validation			
	AUC	Precision	Recall (sensitivity)	F1-score*	AUC	Precision	Recall (sensitivity)	F1-score*
MEWS	0.686 (0.622–0.750)	0.677 (0.612–0.742)	0.705 (0.642–0.768)	0.623 (0.556–0.690)	0.714 (0.672–0.756)	0.703 (0.66–0.746)	0.559 (0.513–0.605)	0.495 (0.448–0.542)
NEWS	0.806 (0.751–0.861)	0.780 (0.723–0.837)	0.783 (0.726–0.840)	0.775 (0.717–0.833)	0.822 (0.786–0.858)	0.772 (0.733–0.811)	0.734 (0.693–0.775)	0.728 (0.687–0.769)
WPS	0.795 (0.739–0.851)	0.808 (0.754–0.862)	0.814 (0.760–0.868)	0.809 (0.755–0.863)	0.795 (0.757–0.833)	0.715 (0.673–0.757)	0.709 (0.667–0.751)	0.706 (0.664–0.748)
AI ECG	0.725 (0.712–0.738)	0.692 (0.670–0.714)	0.719 (0.708–0.730)	0.672 (0.644–0.700)	0.729 (0.724–0.734)	0.673 (0.659–0.687)	0.631 (0.597–0.665)	0.617 (0.564–0.67)
AI ECG with EWS	0.802 (0.798–0.806)	0.762 (0.753–0.771)	0.774 (0.766–0.782)	0.758 (0.750–0.766)	0.833 (0.830–0.835)	0.764 (0.757–0.771)	0.747 (0.741–0.753)	0.747 (0.741–0.753)

Data in parentheses represent 95% confidence intervals.

AI: artificial intelligence; avg., average; ECG, electrocardiography; EWS, Early Warning Scores; MEWS, Modified Early Warning Score; NEWS, National Early Warning Score; WPS, Worthing Physiological Scoring System.

*F1 Score (balanced F-score) is the harmonic mean of precision and recall and was calculated as follows: F1 score = 2 (precision × recall) / (precision + recall).

### AI-ECG as a significant predictor of mortality risk in admission of COVID-19 patients

Table 5 presents the analysis of risk factors associated with mortality in COVID-19 patients during hospitalization. In the Cox proportional hazards models for mortality in the admission of COVID-19 patients, after adjusting for age, sex, and relevant variables, including the EWS systems, the AI-ECG showed a significantly higher hazard ratio of 2.019 (95% CI: 1.156–3.525, *p* = 0.014; Table 5).

**Table 5:** Cox regression analysis for mortality in admission of COVID-19 patients.

	Univariate		Multivariate	
	HR (95% CI)	*p*-value	HR (95% CI)	*p*-value
Age, years	1.087 (1.059–1.117)	< 0.001	1.061 (1.030–1.093)	< 0.001
Male	1.891 (1.171–3.054)	0.009	1.982 (1.205–3.259)	0.007
Heart failure	2.539 (1.259–5.121)	0.009	1.505 (0.705–3.215)	0.291
Hypertension	1.166 (0.733–1.853)	0.516		
Diabetes mellitus	1.229 (0.744–2.031)	0.420		
MEWS	1.348 (1.216–1.494)	< 0.001	1.160 (0.933–1.441)	0.181
NEWS	1.205 (1.132–1.284)	< 0.001	1.213 (0.961–1.531)	0.104
WPS	1.224 (1.131–1.324)	< 0.001	0.831 (0.657–1.050)	0.121
AI ECG	3.230 (1.940–5.378)	< 0.001	2.019 (1.156–3.525)	0.014

AI: artificial intelligence; CI, confidence interval; ECG, electrocardiography; MEWS, HR, hazard ratio; Modified Early Warning Score; NEWS, National Early Warning Score; WPS, Worthing Physiological Scoring System.

### ECG wave analysis using class activation maps

We performed a CAM to demonstrate ECG waveforms for COVID-19 patients throughout severity classifications to better understand the impact of COVID-19 on ECG. As illustrated in the Supplementary Figure, the activation map identified the P wave, the onset of the QRS complex and the T wave as pivotal regions for patients with mild-to-moderate illness, while the QRS complex and the T wave were prominently highlighted for patients with severe-to-critical illness (Supplementary Figure).

## Discussion

We developed a new AI algorithm using initial 12-lead ECGs to identify disease severity and prognosis in patients hospitalized with COVID-19. The algorithm demonstrated reasonable accuracy for internal and external validations. To the best of our knowledge, this is the first study to develop a deep neural network that assesses the severity of COVID-19 based on initial ECGs at admission. Our algorithm can help identify patients who are more likely to develop severe-to-critical illness, thus enabling the effective deployment of medical resources and provision of adequate patient care in the early stages of a large-scale outbreak. Our AI algorithm showed the predictive value of an ECG in identifying COVID-19 severity using a deep learning algorithm. Compared to the previously commonly used physiological scoring systems, the AI-ECG had reliable performance in estimating the severity of COVID-19 in patients. The AI-ECG, combined with the EWS, had a more desirable performance in predicting the severity of COVID-19 (AUC of 0.833 [95% CI: 0.830–0.835], recall of 0.747, F1 score of 0.747, and overall accuracy of 0.745 than that of previous physiological scoring systems.

### Efficient initial patient triage using the AI-ECG

A prior AI model (using a single 12-lead ECG) was created to develop a screening test to exclude those with COVID-19 infection from the general population^2^. Our AI algorithm may support physicians’ decision-making regarding patient referral and assist in screening patients at high risk of progressing to severe disease within the limitations of medical resources. Rapid and accurate point-of-care testing using this AI method can improve patient prognosis by focusing on effective critical care treatment in a limited healthcare system. Furthermore, AI-ECG algorithms have the potential to be applied to recently available smartphones and wearable ECGs. Therefore, AI-ECG provides a fast, reliable, efficient, inexpensive, harmless, and easily accessible method for severity screening and predicting the prognosis of COVID-19. Further, in response to the pandemic, most countries have established community treatment centers for COVID-19 patients or advocated for home isolation to manage medical resources efficiently, particularly regarding bed availability. The rapid clinical deterioration typically experienced by COVID-19 patients, often progressing within a few days from disease onset, underscores the importance of timely transfers from these facilities to hospitals equipped to manage severe to critical conditions^19–21^. The use of relatively simple, non-invasive, and cost-effective examinations, like an ECG, can be advantageous in these circumstances. This study was conducted with the anticipation that this approach would facilitate the efficient allocation of medical resources and consequently improve patient prognoses in upcoming pandemic scenarios similar to COVID-19.

### Impact of COVID-19 on ECG

In this study, patients with severe-to-critical illness had a higher heart rate, prolonged PR interval, QRS duration, and corrected QT interval than patients with mild-to-moderate illness. This may be explained by the effect of coronaviruses on both cardiac function and electrophysiology^22–24^. COVID-19 affects the QT interval independently of factors that may cause QT prolongation; additionally, it is associated with severe cardiac inflammation and renin–angiotensin system activation, known to affect repolarization^18, 23, 25, 26^. Therefore, acute COVID-19 may subtly and pluralistically affect the ECG results^27^. Furthermore, cardiac depolarization and repolarization are complex and delicate processes that can be affected by cardiac dysfunction, metabolic and electrolyte imbalances, and medications, which are factors that affect patients with COVID-19. Moreover, QT prolongation is also a marker of systemic illness severity and increased mortality, as well as an independent risk factor for sudden death both in the general population and those in the ICU^22^.

Previous studies indicate that several ECG changes, such as prolonged PR interval, P wave duration, QT interval, and left ventricular hypertrophy, have been identified in ICU patients who died^28^. Heart failure and asymptomatic severe left ventricular dysfunction have both been successfully detected by deep neural networks based on the ECG^29^. Analyzing ECG waveforms of COVID-19 patients across severity classifications, our CAM analysis revealed distinct patterns. In patients with mild-to-moderate illness, the algorithm highlighted the importance of the P wave, the onset of the QRS complex, and T wave. However, the QRS complex and the T wave emerged as critical areas for those with severe-to-critical disease. Although we cannot fully understand and interpret the decision-making approach in deep learning algorithms due to the “black box” limitation, our results from this analysis support the assumption that ECG changes in mild-to-moderate illness are related to atrial electrical abnormalities, early alterations in ventricular depolarization patterns, and ventricular repolarization abnormalities. Conversely, the severe-to-critical disease exhibited more extensive ventricular depolarization and repolarization abnormalities. These observations suggest atrial and ventricular electrical remodeling and their potential impact on the decision-making process in deep learning algorithms^30^. Thus, such electrocardiographic changes may help with the risk stratification of severity and prognosis in patients with COVID-19.

### AI-ECG and previous early warning scoring systems predict the severity in patients with COVID-19

EWSs are widely used in clinical practice to help doctors estimate the risk of deterioration, monitor the patient's evolution, and make clinical decisions to enhance the critical patient's safety. Many EWS models have been developed, including the NEWS, MEWS, and WPS^31^. These models are based on the effects of COVID-19 on the cardiovascular and pulmonary systems and several extrapulmonary organs^32^. However, limitations in assessing the vital signs, consciousness, oxygen saturation, and other indirect indicators may be overcome by the AI-based approach based on the ECG.

In a recent study, the AUROCs for the NEWS and MEWS in predicting mortality were shown to be 0.809 (95% CI: 0.727–0.891) and 0.670 (95% CI: 0.573–0.767), respectively^31^. We demonstrated a reasonable accuracy of COVID-19 severity prediction in both internal and external validations. In our study, the developed AI using the initial ECG combined with the EWS for detecting severe-to-critical illness in COVID-19 presented a better performance compared with that of the physiologic scoring systems, MEWS, NEWS, and WPS (AUC of 0.833 [95% CI: 0.830–0.835]). In the early stage of COVID-19, ECG-based AI demonstrated better performance in predicting the progression to severe-to-critical illness than the physiologic scoring systems.

This study had some limitations. First, as this was a retrospective study conducted in a single tertiary hospital in Korea, it is necessary to validate the model with patients in other hospitals and countries. A prospective study is warranted to establish the model's usefulness as a new, feasible, and noninvasive screening tool. Second, although we used CAM to visualize ECG waveforms for COVID-19 patients across various severity classifications to understand better COVID-19's impact on ECG, the interpretation of deep learning models and the underlying rationale of AI decision-making remain inherently challenging due to the nature of AI. Third, given the heterogeneity of the patient population, it is possible that the use of drugs that affect the ECG (e.g., antiarrhythmic drugs) may also have affected the network output. Fourth, it remains unclear whether the changes in the ECGs in the presence of a fever or acute respiratory distress associated with the presence of other infectious agents differed from those of COVID-19. Moreover, SARS-CoV-2 is constantly changing. Many notable strains have emerged, including the Alpha, Beta, Delta, and Omicron, and it remains unclear whether COVID-19-related ECG changes differ if the new mutation is more aggressive, highly contagious, vaccine-resistant, can cause more severe illness, or all of the above, compared with the original strain of the virus. Thus, newer variants may require prospective research into what our AI algorithms will accurately predict. Fifth, despite the favorable performance of our deep learning algorithm, overcoming false positives and negatives to identify the optimal treatment and predict the prognosis remains a critical issue. Although it is difficult to fully rely on the AI-ECG, the algorithm could predict disease severity using the initial 12-lead ECG, which is a rapid, simple, and inexpensive point-of-care test. Sixth, utilizing ECGs obtained from local health centers, private clinics, and primary and secondary hospitals might potentially be more closely aligned with the initial onset following a COVID-19 diagnosis. However, almost all patients were rapidly transferred to our hospital's ED without ECGs, resulting in a minimal time discrepancy from disease onset. Seventh, while our research robustly tested our model compared to established ones and used a separate dataset for validation, the single-center nature coupled with challenges from an imbalanced dataset and limited patients underscores the need for a large-scale study. Finally, recent studies have linked COVID-19 exposure to a higher risk of adverse cardiovascular outcomes, even after recovery from acute illness^33, 34^. Consequently, further research with long-term follow-up in patients with COVID-19 complicated with cardiovascular involvement is required to better understand the long-term cardiovascular consequences of COVID-19 on the AI-ECG.

In conclusion, AI using the initial 12-lead ECG demonstrated reasonable performance for predicting COVID-19 severity in hospitalized patients. This AI algorithm could significantly improve COVID-19 severity screening, both efficiently and inexpensively, considering the limited availability of medical resources in a recurrent pandemic.

### Supplementary Information

Supplementary Legends.

Supplementary Figure 1.

## Data Availability

The data collected from the Inha University Hospital during this study were patient data obtained with the institutional review board’s ethical approval. Following the completion of the data use agreement, which specifies that this information cannot be shared, the corresponding author agrees to share de-identified individual participant data, the study protocol, and the statistical analysis plan with academic researchers. The coding used to train the AI model relies on annotation, infrastructure, and hardware and cannot be released.
