# Supplementary material for: Artificial intelligence-enhanced electrocardiography for early assessment of coronavirus disease 2019 severity
Source: Sci Rep. 2023 Sep 13;13:15187. doi: 10.1038/s41598-023-42252-5 (PMC10499801; doi:10.1038/s41598-023-42252-5)
Supplement: Supplementary file 1 — Supplementary Legends. [file 41598_2023_42252_MOESM1_ESM.docx]

**Supplemental Figure.** Class Activation Maps to differentiate COVID-19 severity

**A.** CAM for detecting COVID-19 patients with mild-to-moderate illness

**B.** CAM for detecting COVID-19 patients with severe-to-critical illness

These figures illustrate the activation map, where the prominently bright yellow segments on the waveform indicate regions that have made a substantial contribution to the classification of severity among COVID-19 patients.
