# Supplementary figures and images for: Artificial intelligence-enhanced electrocardiography for early assessment of coronavirus disease 2019 severity
Source: Sci Rep. 2023 Sep 13;13:15187. doi: 10.1038/s41598-023-42252-5 (PMC10499801; doi:10.1038/s41598-023-42252-5)

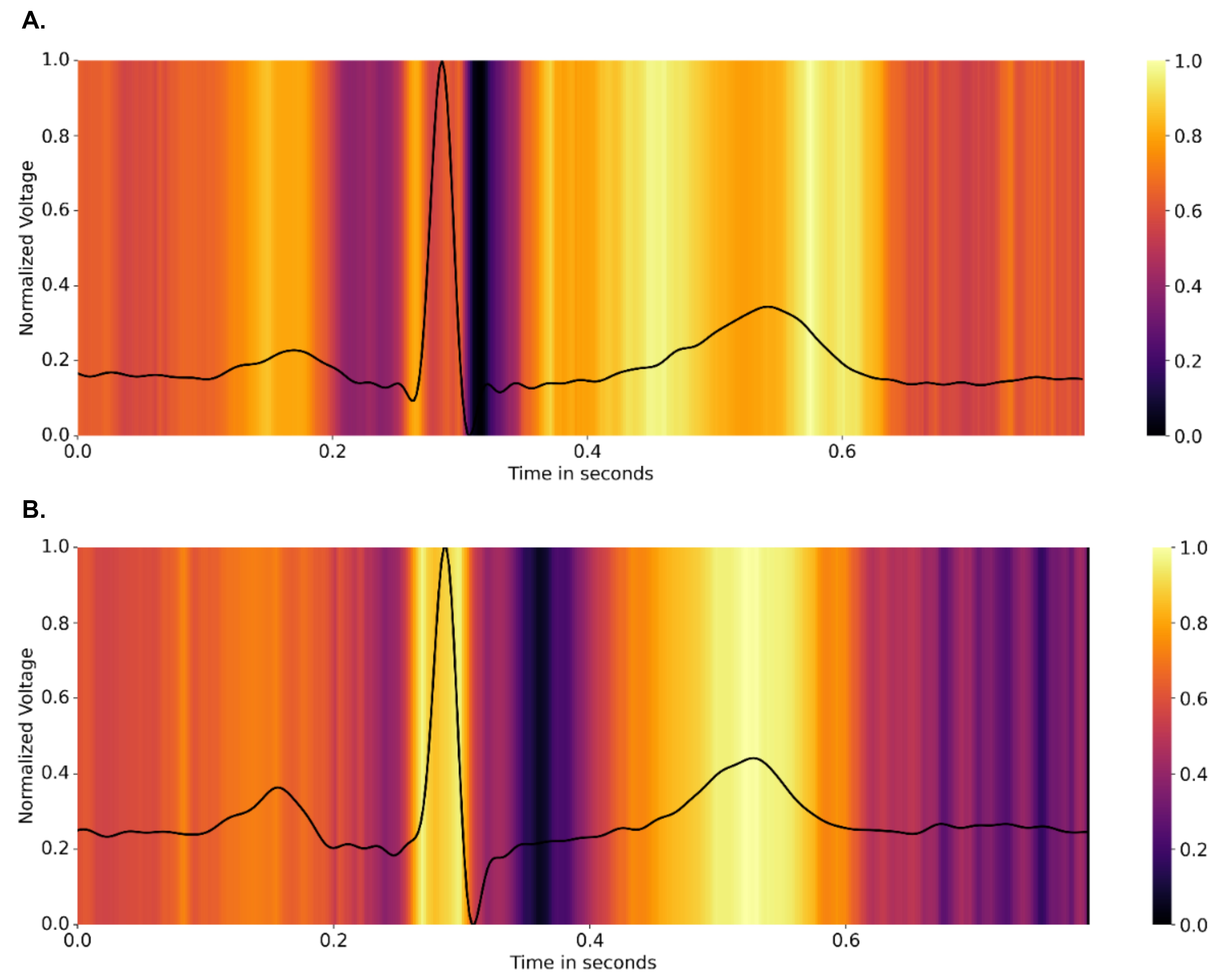

Supplement: Supplementary file 2 — Supplementary Figure 1. [file 41598_2023_42252_MOESM2_ESM.jpg]
